# Demethylation of c-MYB binding site mediates upregulation of *Bdnf IV* in cocaine-conditioned place preference

**DOI:** 10.1038/srep22087

**Published:** 2016-02-25

**Authors:** Weiping Tian, Jiesi Wang, Ke Zhang, Huajing Teng, Chong Li, Moshe Szyf, Zhong Sheng Sun, Mei Zhao

**Affiliations:** 1 Key Lab of Mental Health, Institute of Psychology, Chinese Academy of Sciences, 16 Lincui Rd., Beijing 100101, China; 2 Tianjin Research Center of Basic Medical Science, Tianjin Medical University, Tianjin, 300070, China; 3 Beijing Institutes of Life Science, Chinese Academy of Sciences, Beijing 100101, China; 4 Institute of Genomic Medicine, Wenzhou Medical College, University-town, Wenzhou, Zhejiang, 325000, China; 5 McGill University, Department of Pharmacology and Therapeutics, Montréal, Québec, Canada H3G 1Y6

## Abstract

Abnormal BDNF signaling contributes to the structural and behavioral plasticity induced by drugs of abuse. However, the mechanisms regulating expression of *Bdnf* in drug addiction remain elusive. In the present study, using the conditioned place preference (CPP) model, we showed that expression of *Bdnf IV* is upregulated in the nucleus accumbens (NAc) of conditioned animals while *Bdnf I* is upregulated in cocaine-treated mice irrespective of conditioning. The methylation level of a putative c-MYB binding site in the promoter region of *Bdnf IV* was significantly decreased in the NAc under cocaine CPP conditioning but remained unchanged without conditioning, concurrently with increased binding of c-MYB to this site. Exon IV promoter/luciferase reporter assays revealed that transactivation of *Bdnf* by c-MYB was blocked by methylation of this c-MYB binding site. Administration of methionine, a precursor of SAM, inhibited cocaine CPP, reversed demethylation of c-MYB binding site and induction of *Bdnf IV* expression by cocaine CPP. Our results imply that *Bdnf IV* demethylation at c-MYB binding site is involved in cocaine-triggered seeking behavior, whereas *Bdnf I* responds to the immediate pharmacological effects of cocaine.

Brain-derived neurotrophic factor (BDNF) plays a crucial role in synaptic plasticity and memory processing in the adult brain^1^. Abnormal BDNF signaling is hypothesized to contribute to the structural and behavioral plasticity induced by drugs of abuse^2^. It has been reported that the levels of *Bdnf* mRNA are significantly increased in the nucleus accumbens (NAc) following acute and repeated cocaine treatment, as well as during cocaine withdrawal^3^. Additionally, the BDNF protein is transiently elevated in NAc of rats following intravenous cocaine self-administration^4^. Furthermore, local injection of BDNF into the NAc or ventral tegmental area (VTA) enhances locomotor sensitization to cocaine, potentiation of cocaine-related conditioned reward, and drug seeking after cocaine withdrawal^5^. Conversely, NAc infusion of antibodies directed against BDNF or TrkB (the receptor for BDNF) inhibits methamphetamine-induced dopamine release and locomotor behavior^6^. As the dopaminergic projection from the VTA to the NAc contributes to reward and reinforcement associated with drug addiction, the level of NAc BDNF is suggested to play an important role in cocaine rewarding effects^7^.

The *Bdnf* gene is regulated by at least eight discrete promoters that initiate transcription of multiple distinct *Bdnf* mRNAs from different unique sites, each of which encodes an identical protein^8^,^9^. The distinct physiological role of each of the different *Bdnf* splice variants is still unknown. It has been reported that acute but not chronic cocaine treatment increases the expression of the *Bdnf IV* (formerly characterized as *Bdnf III*) splice variant in the striatum^10^, suggesting that distinct sets of *Bdnf* promoters differentially control *Bdnf* production, and that each of the splice variants is restricted to distinct biological functions, such as drug addiction.

Although the regulatory mechanisms that mediate changes in expression of *Bdnf* and its splice variants in response to different stimuli are unclear, epigenetic modifications, such as DNA methylation, have been shown to play at least a partial role. It has been reported that increased synthesis of *Bdnf* in cultured cortical neurons in response to membrane depolarization is correlated with decreased CpG methylation within a regulatory region of the *Bdnf* gene^11^. Although accumulated evidence strongly supported the roles of DNA methylation in cocaine addiction^12^,^13^,^14^,^15^, it is still unknown whether DNA methylation plays a role in controlling splice-variant specific transcription of *Bdnf* splice forms in cocaine rewarding memory.

In this study, we investigated the effects of cocaine conditioned place preference (CPP) training on DNA methylation status in the promoter regions of different *Bdnf* splice variants in the NAc and established a correlation between DNA methylation at –148 sites of *Bdnf IV* promoter and expression of the *Bdnf IV* splice variant. Finally, to determine whethter a causal relationship exists between methylation and expression of *Bdnf IV*, we measured the effects of treatment with methionine, a methyl group donor, on DNA methylation of the *Bdnf IV* promoter, *Bdnf IV* expression *and* cocaine CPP.

## Results

### Regulation of different Bdnf splice variants by cocaine and food reward

**Figure 1:**
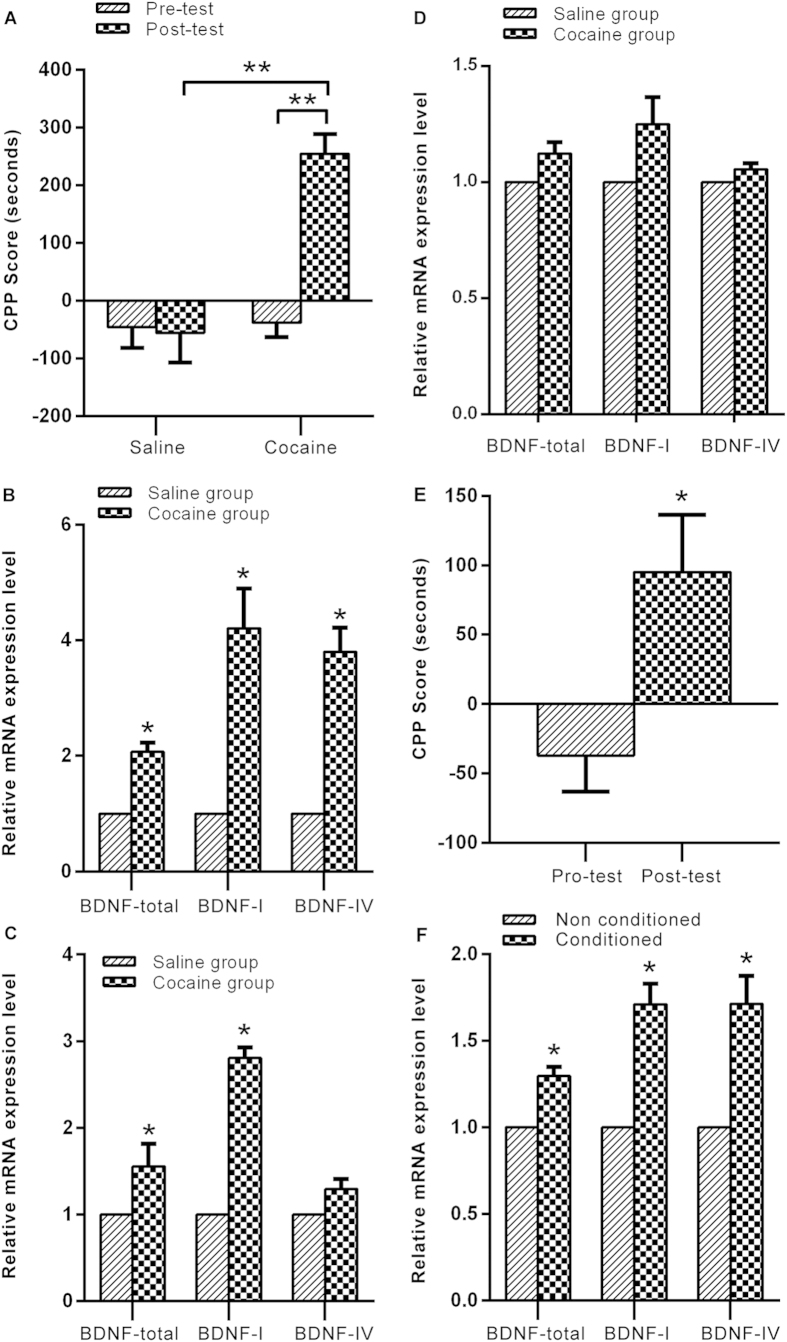
Changes in total *Bdnf*, *Bdnf I* and *Bdnf IV* expression after cocaine and food CPP training. (**A**) Acquisition of cocaine CPP. (**p < 0.01, saline group n = 13, cocaine group n = 20). (**B,C**) Changes in total *Bdnf*, *Bdnf I* and *Bdnf IV* expression in the NAc in cocaine-conditioned-mice (**B**) and non-conditioned (**C**). (**D**) Changes in total *Bdnf*, *Bdnf I* and *Bdnf IV* expression in the PFC in cocaine-conditioned-mice. (**E**) Acquisition of food CPP. (*p < 0.05, n = 19) (**F**) Changes in total *Bdnf*, *Bdnf I* and *Bdnf IV* expression in the NAc in Food-conditioned-mice. mRNA expression data are depicted as the relative gene expression level in the treatment group over the relative gene expression in the control group, *p < 0.05, triplicate in RT-qPCR repeat.

As shown in Fig. 1A, two-way ANOVA revealed that CPP score was significantly increased [Treatment: F_(2,49)_ = 7.9, *p* = 0.001; Test: F_(1,49)_ = 29, *p* < 0.001; Interaction: F_(2,49)_ = 11, *p* < 0.001] after cocaine CPP training. The mRNA levels of total *Bdnf* and *Bdnf I* were upregulated significantly in both conditioned (t = 6.75, *p* < 0.05 for total *Bdnf* and t = 4.67, *p* < 0.05 for *Bdnf I*) and non-conditioned mice (t = 9.43, *p* < 0.02 for total *Bdnf* and t = 14.67, *p* < 0.005 for *Bdnf I*), while the mRNA level of *Bdnf IV* was upregulated only in conditioned (t = 6.68, *p* < 0.05) but not in non-conditioned animals (Fig. 1B,C). The levels of expression of total *Bdnf*, *Bdnf I* or *Bdnf IV* remained unchanged in the prefrontal cortex (PFC) in conditioned mice (Fig. 1D).

As shown in Fig. 1E, the CPP score was significantly increased (t = 2.72, *p* = 0.01) after 8 days of alternate pairing with food and no food. Figure 1F shows that the mRNA levels of total *Bdnf* (t = 49.46, *p* < 0.001), *Bdnf I* (t = 4.54, *p* < 0.05) and *Bdnf IV* (t = 11.19, *p* < 0.01) were upregulated in the NAc after food CPP training.

### Demethylation of c-MYB binding site and increased binding of c-MYB mediates upregulation of Bdnf IV after cocaine CPP conditioning

**Figure 2:**
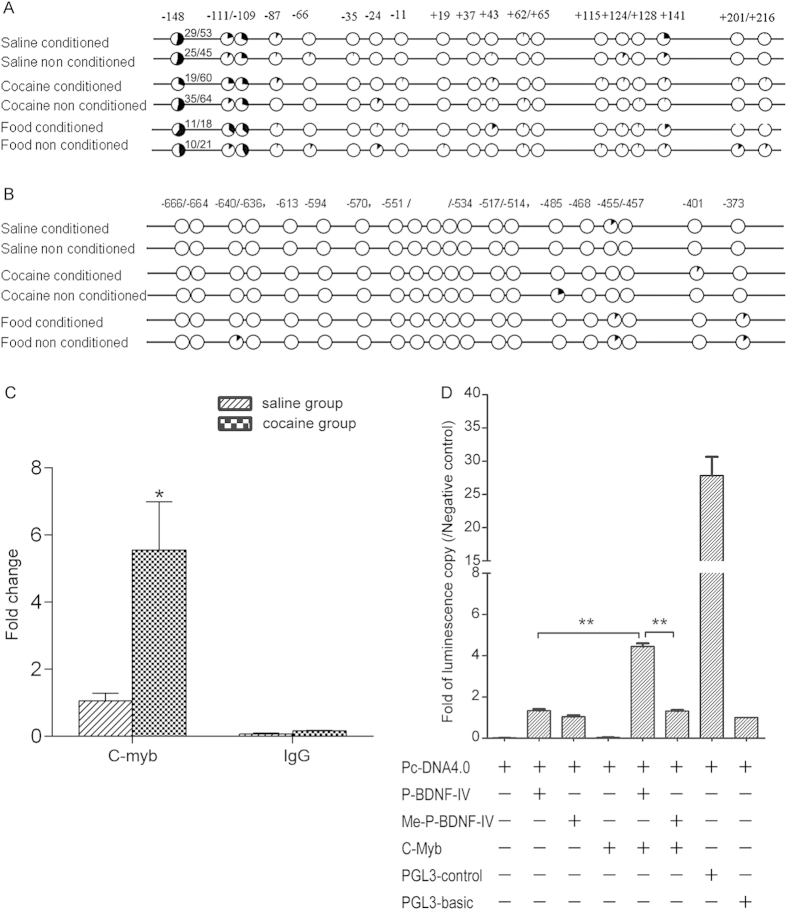
(**A**) DNA methylation levels of the promoter regions of *Bdnf* *IV* in mouse NAc. Fraction of methylated clones at each CpG site within the promoter of *Bdnf IV* for saline-conditioned (Sc), saline non-conditioned (Sn), cocaine-conditioned (Cc), cocaine non-conditioned (Cn), food-conditioned (Fc) and food non-conditioned (Fn) group. The data is presented as the number of methylated clones over the total number of clones at each CpG site (“number of methylated CpG at -148/number of total sequenced clones” is shown at the right of the -148 plots). (**B**) Percentage of methylated clones at different CpG sites upstream of the transcriptional start site of *Bdnf I* for Sc, Sn, Cc, Cn, Fc and Fn group. (**C**) The status of c-MYB binding in *Bdnf IV* promoter (*p < 0.05, saline vs. cocaine group, triplicate in qPCR repeat). (**D**) Luciferase expression in the presence or absence over expression of c-MYB. Data depicted as the relative ratios of luciferase activity to the negative control, **p < 0.01, P-*Bdnf*-IV: *Bdnf IV* promoter luciferase construct; Me-P-*Bdnf*-IV: *Bdnf IV* promoter luciferase construct with methylated MYB binding site; c-MYB: mouse c-MYB expression construct; positive control: PGL3-control vector; negative control: PGL3-basic vector.

To investigate a role of DNA methylation in cocaine CPP, we examined the levels of methylation of candidate cytosine sites in promoter regions from base pair –691 to –377 for *Bdnf I and* from –230 to +248 for *Bdnf IV*, using sequencing of several individual clones amplified from bisulfite-converted DNA. We selected these regions according to previous studies in which DNA methylation changes within these regions were shown to be involved in regulating *Bdnf I*^16^ or *Bdnf IV*^17^. Results from at least three independent experiments showed that a single cytosine site at –148 within the promoter region of *Bdnf IV* was significantly hypomethylated after conditioned cocaine CPP but not after saline CPP (the methylation levels were 54.7%, 55.6%, 31.7% and 54.7% in saline conditioned (Sc), saline non-conditioned (Sn), cocaine-conditioned (Cc) and cocaine non-conditioned (Cn) groups, respectively; Sc vs. Cc, *p* < 0.05; Fisher’s exact test) (Fig. 2A). In addition, neither conditioned nor non-conditioned food CPP induced DNA methylation status alteration at this site (61.1% and 47.6%, respectively). As shown in Fig. 2B, neither cocaine nor food treatment altered the level of DNA methylation in *Bdnf I* in the NAc.

Using TFSEARCH (http://www.cbrc.jp/research/db/TFSEARCH.html), we found that the hypomethylated site at –148 in the *Bdnf IV* promoter was a potential binding site for c-MYB, a nuclear DNA-binding protein that recognizes a consensus sequence motif and regulates the activity of promoters that contain this motif^18^. A chromatin immunoprecipitation (ChIP) assay using an anti-MYB antibody revealed that the binding of c-MYB was significantly increased in cocaine conditioned group compared to saline conditioned group (t = 3.11, *p* < 0.05) (Fig. 2C). Figure 2D showed that transcriptional activity of the unmethylated *Bdnf IV* promoter as measured in a transient transfection luciferase reporter assay was increased by 3-fold in the presence of ectopically expressed *c-Myb* expression vector, whereas this did not occur with the methylated vector (*p* < 0.001).

Considering the results of the luciferase reporter assay which demonstrated that the methylation status of the predicted binding site of c-MYB affects promoter activity and the fact that CpG methylation affects the binding of transcription factors to the promoter region, we reasoned that this effect may be mediated by c-MYB binding to this site which is consistent with our ChIP data. These results suggest that methylation of the –148 cytosine can suppress c-MYB transactivation of *Bdnf IV* by inhibiting binding to its DNA binding motif, which likely contains the −148 cytosine locus.

### Systemic administration of methionine attenuated the CPP expression of cocaine, reversed the induction of *Bdnf IV* transcription and demethylation of c-MYB binding site

**Figure 3:**
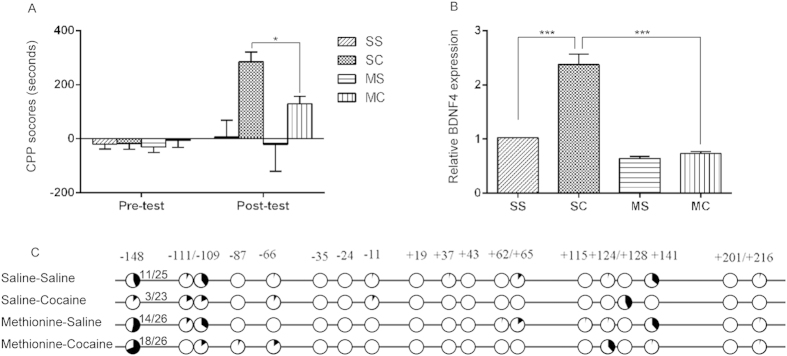
The effects of chronic methionine treatment on the expression of cocaine-CPP, *Bdnf* IV expression and methylation level of its promoter. (**A**) methionine treatment significantly attenuated the expression of cocaine-CPP (*p < 0.05, n = 8~10 for each group); (**B,C**) methionine treatment reversed the induction of *Bdnf* IV transcription (*p < 0.001, triplicate in RT-qPCR repeat) (**B**) and demethylation of c-MYB binding site (**C**) by cocaine CPP. (SS: Saline-Saline; MS: Methionine-Saline; SC: Saline-Cocaine; MC: Methionine-Cocaine. (“number of methylated CpG at −148/number of total sequenced clones” is shown at the right of −148 plots).

If DNA methylation is indeed causal in *Bdnf IV* expression and the CPP phenotype, then pharmacological manipulation by “epigenetic” drugs should alter its expression and reverse CPP behavior. We used methionine, a precursor of S-adenosyl methionine which is a ubiquitous methyl donor and has been previously used to increase DNA methylation in the brain^19^. As shown in Fig. 3C, methionine reversed, the reduction in DNA methylation at −148 in the *Bdnf IV* promoter that has been induced by cocaine CPP (SS: 44%; MS: 53.8%; SC:13.0%; MC:69.2% respectively; SS vs. SC, *p* < 0.05; SC vs. MC, *p* < 0.001; Fisher’s exact test) (Fig. 3C). The induction of *Bdnf IV* mRNA by cocaine was significantly decreased by co-administration of methionine (F_(3,8)_ = 40.68, *p* < 0.001; S-C vs. M-S, *p* < 0.001) (Fig. 3B) and methionine administration significantly attenuated cocaine-CPP behaviors (F_(3,32)_ = 4.3, *p* < 0.05; SC vs. MC, *p* = 0.029) Fig. 3A.

## Discussion

The current study demonstrated that expression of total *Bdnf* and *Bdnf I* was upregulated in both cocaine-conditioned and non-conditioned mice, whereas the expression of *Bdnf IV* was only upregulated in the NAc, and only in conditioned mice. Our findings suggest that *Bdnf IV* might contribute to cocaine reward seeking behavior, whereas *Bdnf I* responds to the direct pharmacological effects of cocaine. These conclusions were similar to those of a previous study that used the contextual fear conditioning model and showed that only *Bdnf IV* was transcriptionally upregulated in the hippocampus during consolidation of fear learning, whereas *Bdnf I* but not *Bdnf IV* was upregulated after the same context exposure alone^20^. We believe that a differential, splice-variant initiation of *Bdnf* transcription exists in context exposure versus associative contextual conditioning in specific brain regions.

To date, several transcription factors have been reported to contribute to the regulation of *Bdnf* transcription by binding to specific sites in the *Bdnf* promoter regions such as cAMP-responsive element binding protein, upstream stimulatory factors and calcium-responsive transcription factor^21^,^22^,^23^,^24^. Our study identified a new transcription activator for *Bdnf*: c-MYB. Previous studies have shown that c-MYB mostly operates as a transcriptional activator by binding to the sequence t/cAACt/gG, which is known as the MYB binding site^18^. It also cooperates with members of the CCAAT-box enhancer-binding protein family to active mim-1 gene^25^. The constitutive and aberrant expression of c-MYB in normal and Cu/Zn SOD mutant mouse brain has been reported previously, suggesting that c-MYB plays a role in normal physiology as well as pathological states^26^,^27^. Here, we show that the induction of *Bdnf IV* transcription was mediated by increased binding of c-MYB, a transcription factors, to its binding site in *Bdnf IV* promoter in chromatin *in vivo* which was demethylated by cocaine CPP conditioning. It should be noted however that further *in vitro* experiments are necessary to characterize and demonstrate direct interaction between c-MYB and the −148 putative c-MYB recognition element. The role for demethylation in upregulating *Bdnf IV* in response to cocaine triggered CPP was supported by further results showing that methionine inhibited cocaine CPP, concurrently with reversal of demethylation of c-MYB binding site and the induction of *Bdnf IV* expression. Methionine is a general modulator of the supply of methyl moieties for methylation reactions in cells and might affect other methylation reactions. Nevertheless, it is interesting to note that we observe specificity for the −148 CG site which is probably more prone to become methylated when general methylation conditions are altered in the NAc. Thus, our data is consistent with the hypothesis that methylating the −148 site reduces expression of the *Bdnf* gene. However, our data does not exclude other methylation events that might be of importance as well.

To the best of our knowledge, this is the first report of a transcriptional regulatory relationship between c-MYB and *Bdnf IV*. We believe that systematic analysis of the target genes regulated by c-MYB might provide further insight into the understanding of the novel biological functions of c-MYB in psychiatric disorders.

Interestingly, DNA demethylation of a specific site could active *Bdnf IV* expression in mice that were trained to cocaine CPP, but not in mice that were only injected with cocaine. These results suggest that the pharmacological effects of cocaine are not sufficient to alter DNA methylation status at this special site, and a pairing between cocaine and a cue is necessary to induce this epigenetic modification. It is found that the levels of *Bdnf IV* transcripts show a longer-lasting elevation than *Bdnf I* after kainic acid treatment^9^, which implies that *Bdnf IV* might exert longer-lasting effects on those events that involve changes in BDNF expression. Drug addiction is considered to be a type of aberrant learning and memory that involves long-lasting neural plasticity in brain reward regions^28^,^29^. Importantly, the level of BDNF protein in the NAc progressively increases during cocaine withdrawal^4^. Together, the induction of *Bdnf* especially *Bdnf IV* may represents a stable neuroplasticity candidate biomarker for transition from social drug use to regulated and compulsive relapse^30^. This may be due in part to the persistent activation of its transcription by DNA demethylation at regulatory regions as it is believed that DNA methylation could exert long-lasting control over gene expression, and can mediate stable changes in brain function^31^.

Previous studies demonstrated that anterograde transport is important for the trafficking of BDNF in the central nervous system and that trafficking of BDNF in the striatum from the PFC has clear functional roles^32^. However, local expression of *Bdnf* is dramatically enhanced in the striatum under pathological conditions^33^. Moreover, downregulation of endogenous *Bdnf* using viral-mediated siRNA injection into the striatum significantly increased ethanol self-administration, suggesting a role for locally expressed striatal *Bdnf*^34^. We didn’t find significant changes of *Bdnf* mRNA expression after cocaine CPP in the PFC, indicating that changes in mRNA expression of *Bdnf* in the NAc did not derive from changes in *Bdnf* levels in the PFC. Taking these together, we suggest that local changes in expression of *Bdnf IV* in the NAc that are triggered by cocaine CPP may have functional roles.

Another question raised by the current study is why “food CPP” conditioning did not result in demethylation of the promoter region of *Bdnf IV*, although *Bdnf IV* expression showed a significant increase in response to food CPP conditioning. In fact, both drugs of abuse and food increase NAc dopamine level, but the dopamine response to drugs of abuse does not habituate to the same extent as it does to food^35^. This could be supported by evidence that cocaine but not food or sucrose reward self-administration produce persistent potentiation of VTA excitatory synapses, even when drug-seeking behavior is extinguished^36^. Some recent studies have shown that a different epigenetic mechanism, histone acetylation, is involved in regulating gene transcription in cocaine but not sucrose self-administration^37^. CPP training in response to cocaine, but not to food, decreased global DNA methylation while chronic treatment of methionine attenuated the expression of CPP but did not affect the establishment of food-CPP^12^. Together with these previous data, we suggest that epigenetic modifications might have induced a transcriptional program that led to long-lasting activation of plasticity-related genes that underlie the long-lasting adaptation induced by drug-rewarding memory. In contrast, natural rewards that are fundamental for survival are less prone to result in lasting modification by external stimuli.

Relapse is a major challenge in the treatment of drug addiction, partly due to the difficulties in extinguishing the ability of drug-associated cues to trigger craving and relapse^38^. The current study demonstrates that DNA demethylation of c-MYB binding site could activate *Bdnf IV* expression in mice that were trained for cocaine CPP, but not in mice that were only injected with cocaine. Although many questions remain to be answered with regard to the long-lasting regulatory mechanisms of different *Bdnf* variants that underlie drug addiction, our results suggest that blocking the association between drug and cues through reversal of DNA demethylation is a potential novel approach for treatment of cocaine addiction.

## Materials and Methods

### Animals

Adult male C57/BL6 mice (20–30 g) were housed individually under a 12 h light/dark cycle with supplementary of food and water *ad libitum* except for mice trained for food CPP. All protocols were approved by the Review Board of the Institute of Psychology, Chinese Academy of Sciences [protocol number: A12031, May 2012] and were performed strictly in accordance with the Guideline for Care and Use of Laboratory Animals of the Chinese Academy of Sciences.

### Conditioned place preference (CPP) procedures and methionine treatment

An unbiased conditioning protocol of CPP was used as described previously^39^, which consisted of three different phases: habituation (pre-conditioning), conditioning (8 sessions), and drug-free test (post-conditioning). On the pre-conditioning day, mice were placed in the center of a three-chamber box and were allowed to roam freely between the two chambers for 900 sec. During the conditioning phase of cocaine CPP, animals were paired for 8 days with the saline group receiving saline in both sides of the chambers, and drug groups receiving cocaine (20 mg.kg.i.p.) on one side and saline on the opposite side^40^. For training of food CPP, mice were individually housed with free access to drinking water but limited access to 2.2–2.5 g lab chow per day for 7 days before training, resulting in a loss of 15% of the original body weight. During the conditioning phase, the mice received food on one of the sides and received nothing on the opposite side. After each injection (the mice in the food CPP groups were not injected), the mice were confined to the corresponding conditioning chambers for 30 min for cocaine CPP or 50 min for food CPP before returning to their home cages. On the test day, the mice were allowed to roam freely between the two sides. The place preference score (CPP score), was defined as the time spent in the cocaine- or food-paired chamber minus the time spent in the cocaine- or food-unpaired chamber^41^. The non-conditioned animals were administrated 8 consecutive days with alternate injections of cocaine and saline in their home cages but not confined to either chamber. To exclude the effects of food restriction, non-conditioned animals were food restricted but were not trained for food CPP.

For methionine (Sigma, St. Louis, MO, USA) treatments, mice were injected subcutaneously with 1 g/kg (6.6 mmol/kg) L-methionine twice a day for 15 and 10 consecutive days before and during CPP training respectively. This was the effective dose and duration shown to increase methylation level at DNA in certain regions in the brain^42^.

### RNA isolation and real-time PCR

All NAc tissues were collected at 2hr after CPP test. Total RNA and DNA were extracted using DNA/RNA isolation kit (Tiangen Biotech, Beijing, China) following the manufacturer’s instructions, and 1.0 μg total RNA was reverse-transcribed into single-strand cDNA using Superscript III (Invitrogen, Carlsbad, CA, USA). Real-time PCR was performed on an Opticon 2 real-time PCR machine (Rad, Hercules, CA, USA) using SYBR Green mix (Tiangen) with the following cycling program: 95 °C for 10 min, 40 cycles of 95 °C for 25 s, 60 °C for 25 s, and 72 °C for 25 s. An aliquot of cDNA was amplified with using a pair of primers as shown in Table 1. GAPDH was used as an internal control for normalization. Each sample was tested in triplicates.

### Bisulfite sequencing and analysis of the DNA methylation levels

Genomic DNA (**7**50 ng) was treated with EZ DNA Methylation-Direct^TM^ Kit (Zymo Research, Irvine, CA, USA) which converts all non-methylated cytosine into uracil while leaving 5-methylcytosines unaltered following the manufacturer instructions. The converted DNA fragments were then used to amplify the region within the *Bdnf I and IV* promoters using nested PCR. The sequences of the primer sets were as follows:

For *Bdnf I* outer primers: 5′-AGGATTGGGTGGTTTAAAATTTTAG -3′, 5′-AACTTTCCCTTTTCCTCTTAAAAACTA-3′;

Nested primers: 5′-TATTAGTGGGAAGTGTAGTGGTTAG -3′ 5′-AATTACCCACAAAAACCTATACAAAC-3′.

For *Bdnf IV*, outer primers: 5′-TGTGTTGTTGTTGTTTAGATAATGATAG-3′, 5′-TAAAACCATATACTTCCCAACAAAC-3′

Nested primers: 5′-TAATGATAGGTTTGGTTTTTGTGTG-3′,

5′-TCCCCTTCTCTTCAATTAAAAAAA-3′.

PCR products were gel-purified and were then ligated to pGEM-T Vector (Promega, Madison, WI, USA). Following transformation, at least 20 clones for each group (for cocaine CPP groups and its control, clones number = 45–64) were sequenced at the Beijing Genomics Institute (BGI, Beijing, China). Sequencing results were analyzed on line at http://quma.cdb.riken.jp/.

### Myb expression plasmid construction

Mouse cDNA was used as a PCR template to generate the full length *Myb* cDNA with the following primers: 5′AAT AAG CTT ATG GCC CGG AGA CCC CGA C 3′ and 5′ CC CTC GAG TCA CAT GAC CAG AGT TCG AGC T 3′. Following standard ligation and subcloning, the PCR product was cloned into the Hind III/XhoI sites of the pcDNA4 vector (Invitrogen). The identity of the clone was confirmed by sequencing.

### Generation of constructs with site-specific methylation

Luciferase reporter construction *of Bdnf* exon IV Promoter **(911 bp)** was a gift from Dr. Wang Hongbin in Department of Physiology and Neuroscience, Michigan State University. Site specific methylation within the gene promoter luciferase construct at the -148 site of upstream of transcript start site in *Bdnf IV* was generated using modified oligonucleotides (SBS, Genetech Co., Beijing, China). The modified oligonucleotide contained a −148 site specific methylated or an unmethylated CpG dinucleotide and spanned 15 bps on either side of the site. PCR was carried out according to the protocol in the Muta-directed^TM^ mutagenesis kit (SBS, Genetech Co.). Following PCR, the PCR mixture was treated with Mutazyme^TM^ for two hours to ensure that the plasmid used for template was digested. The treated products were purified using the Qia-Quik (Qiagen, Hilden, Germany) according to the manufacturer’s protocol. The products were then used directly for transfection into 293 T cells.

### Transient transfections and reporter assays

293 T cells were plated at a density of 2 × 10^5^ in a 12-well dish and were maintained as a monolayer in DMEM/High glucose containing fetal calf serum (10%, Thermo Fisher, Waltham, MA, USA). *c-Myb* expression plasmids were co-transfected with methylated and unmethylated exon IV promoter luciferase constructs, respectively. The transfected 293 T cells were harvested and lysed 24 h after transfection and luciferase activity was assayed using the Promega luciferase assay system. Transfections were repeated three times. The PGL3 control plasmid and PGL3 basic plasmid were used as positive and negative controls, respectively.

### Quantitative chromatin immunoprecipitation (ChIP) assay

NAc tissue samples were treated with the Magna CHIPTM G Tissue kit (Millipore, Billerica, MA, USA) according to the manufacturer’s protocol and sonicated using a Biorupter Pico instrument (Diagenode, Seraing, Belgium) for 12.5 min (45 s on, 30 s off, 10 cycles). Cross-linked chromatin was immunoprecipitated with anti-c-MYB antibody (sc-7874, Santa Cruz Biotechnology, Santa Cruz, CA, USA) targeting its binding site. Normal rabbit IgG-B (sc-2763; Santa Cruz Biotechnology) was used as a negative control antibody. Q-PCR was used to detect the c-MYB occupancy on the ChIPed *Bdnf IV* promoter. The primer set was:

5′-GCGCGGAATTCTGATTCTGGTAAT-3′

5′-GAGAGGGCTCCACGCTGCCTTGACG-3′

### Data analysis

All data are displayed as the mean ± S.E.M. The cocaine CPP scores were analyzed using two-way repeated ANOVAs followed by LSD post-test with the between-subjects factors of treatment (saline or cocaine) and the within-subjects factor of test condition (pre-conditioning or post-conditioning), and the food CPP scores were analyzed using t-test. The expression levels of genes were analysed using One-sample t tests or one-way ANOVAs. The one-way ANOVAs were used to analyze luciferase activity. The methylation levels at different “CG” sites were analyzed on line at http://quma.cdb.riken.jp/. Significance was set at *p* < 0.05 for all data.

## Additional Information

**How to cite this article**: Tian, W. *et al.* Demethylation of c-MYB binding site mediates upregulation of *Bdnf IV* in cocaine-conditioned place preference. *Sci. Rep.* **6**, 22087; doi: 10.1038/srep22087 (2016).

## Figures and Tables

**Table 1:** Sequence of primers used for RT-PCR.

Gene	Sequence of primer (5′-3′)	
	Forward	Reverse
*Bdnf*-total	TGGCTG ACACTTTTGAGCAC	AAGTGTACAAGTCCGCGTCC
*Bdnf I*	AGTCTCCAGGACAGCAAAGC	ACACCTGGGTAGGCCAAGTT
*Bdnf IV*	CAGAGCAGCTGCCTTGATGTT	GCCTTGTCCGTGGACGTTTA
*Gapdh*	TGCACCACCAACTGCTTA	GGATGCAGGGATGATGTTC
